# Acceptance of animal research in our science community

**DOI:** 10.12688/f1000research.8169.2

**Published:** 2016-07-08

**Authors:** Konstantin Bergmeister, Bruno Podesser

**Affiliations:** 1 CD Laboratory for the Restoration of Extremity Function, Department of Surgery, Medical University of Vienna, Vienna, Austria; 2 Department of Biomedical Research, Medical University of Vienna, Vienna, Austria

**Keywords:** Animal research, survey, acceptance animal research, Stop vivi-section

## Abstract

Animal research is debated highly controversial, as evident by the “Stop Vivi-section” initiative in 2015. Despite widespread protest to the initiative by researchers, no data is available on the European medical research community’s opinion towards animal research. In this single-center study, we investigated this question in a survey of students and staff members at the Medical University of Vienna. A total of 906 participants responded to the survey, of which 82.8% rated the relevance of animal research high and 62% would not accept a treatment without prior animals testing. Overall, animal research was considered important, but its communication to the public considered requiring improvement.

## Introduction

Animal research is still debated, highly controversial, and lately has attracted great attention as over 1.1 million European citizens signed the “Stop Vivi-section” initiative in 2015, demanding the stop of all animal research ^1^. Alarmed by the potential consequences opinion leaders made efforts to illustrate the need for animal experiments for medical progress ^2, 3^. However, does the European medical research community stand united behind animal research?

## Methods

In an internal survey at the Medical University of Vienna we investigated the positions towards animal research of 10335 (M.D. and Ph.D.) students and 3824 medical staff members. The survey was conducted using the MedCampus system (CAMPUSOnline, Graz, Austria) of the Medical University of Vienna, accessible to all students and staff members. The survey was conducted over a period of four weeks in November 2015. Statistical analyses were conducted using SPSS (V.21, IBM Corp, US).

**Ethics committee approval:** Approval was obtained from the Medical University of Vienna’s data privacy committee.

## Results

Word file containing survey questions in original German language and translated to English Click here for additional data file. Copyright: © 2016 Bergmeister K and Podesser B 2016 Data associated with the article are available under the terms of the Creative Commons Zero "No rights reserved" data waiver (CC0 1.0 Public domain dedication).

Excel file containing anonymized responses to the survey Click here for additional data file. Copyright: © 2016 Bergmeister K and Podesser B 2016 Data associated with the article are available under the terms of the Creative Commons Zero "No rights reserved" data waiver (CC0 1.0 Public domain dedication).

**Figure 1.**
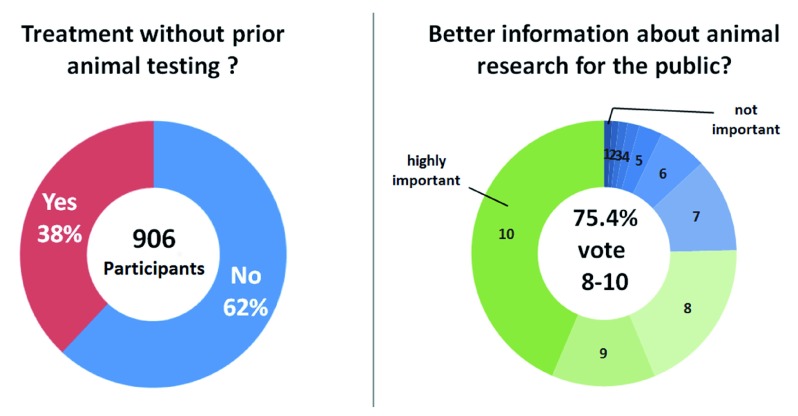
Survey results. Left: A majority of participants would not accept a treatment that has not been previously tested in animal models. Right: The need for better information about animal research for the public was rated high by 75% of the participants.

A total of 906 participants responded to the survey, representing a response rate of 6.38%. Participants were 36.5% staff members and 63.5% students, of which 43% previously had personal experience with animal experiments. The relevance of animal models for research was rated high (8–10 on a scale 1–10; 1 being lowest) by 82.8%, and 62% would not accept a treatment without prior animals testing ( Figure 1, left). These results were similar to a 2011 Nature poll ^4^ with 980 participants and a 2014 survey by the American Association for the Advancement of Sciences ^5^. In our cohort, participants rated society’s acceptance of animal research low (4.24±1.77, scale 1–10; 1 being lowest) as well as the current communication to the public on medical advances derived from animal research (4.37±2.22, scale 1–10; 1 being lowest). Consequently, 75.4% believed the public should receive better information about the benefits, necessities and legislation of animal experiments ( Figure 1, right).

## Discussion

In this study, we assessed the opinions of our faculty members and students towards animal research. Overall, our study population considered animal research important for medical progress. In addition, we see a clear mission to improve communication to the public about animal experiments. Moreover, scientists need to improve the communication of complex results into a language that is understood by society and colleagues alike. Limitations of this study were the small number of participants and being a single-center survey. A comparable nature study ^4^ from 2011 had a relatively lower response rate (approximately 4.9%) and a similar total number of 980 participants.

In conclusion, this single-center study provides first survey results of students and medical faculty members towards animal research. Based on the interesting results, we plan to extend this study to other institutions and thereby provide an overview of the European medical community’s opinion towards animal research.

## Data availability

The data referenced by this article are under copyright with the following copyright statement: Copyright: © 2016 Bergmeister K and Podesser B

Data associated with the article are available under the terms of the Creative Commons Zero "No rights reserved" data waiver (CC0 1.0 Public domain dedication). 

*F1000Research*: Dataset 1. Word file containing survey questions in original German language and translated to English, 10.5256/f1000research.8169.d115219 ^6^

*F1000Research*: Dataset 2. Excel file containing anonymized responses to the survey, 10.5256/f1000research.8169.d115220 ^7^
