# Supplementary material for: Acceptance of animal research in our science community
Source: F1000Res. 2016 Jul 8;5:282. Originally published 2016 Mar 4. [Version 2] doi: 10.12688/f1000research.8169.2 (PMC4866631; doi:10.12688/f1000research.8169.2)
Supplement: Word file containing survey questions in original German language and translated to English [file f1000research-5-9461-s0000.tgz › f7fa1501-62e1-4904-806c-6dee16fd5756_Questions_on_Animal_research.docx]

**Questions on Animal research – Podesser Bergmeister**

1. Question:

**German**: Haben sie persönliche Erfahrungen mit Tierversuchen?

- 1. Ja
  2. Nein

**English**: Do you have personal experience with animal experiments?

1. Yes
2. No
3. Question:

**German:** Würden sie einer medizinischen Therapie zustimmen, die zuvor niemals an einem Tierversuch getestet wurde?

1. Ja
2. Nein

**English:** Would you accept a medical therapy, that has previously not been tested in animal models?

1. Yes
2. No
3. Question:

**German**: Wie bewerten sie die Relevanz von Tierversuchen für die medizinische Forschung?

1 2 3 4 5 6 7 8 9 10

(nicht relevant) (sehr relevant)

Oder: weiß nicht (0)

**English**: How do you rate the relevance of animal experiments for medical research?

1 2 3 4 5 6 7 8 9 10

(not relevant) (very relevant)

Alternative: Don’t know (0).

1. Question:

**German**: Wie bewerten sie die Akzeptanz von Tierversuchen in der allgemeinen Bevölkerung?

1 2 3 4 5 6 7 8 9 10

(keine Akzeptanz) (sehr gut akzeptiert)

Oder: Weiß nicht (0)

**English**: How do you rate the acceptance of animal research in the general public?

1 2 3 4 5 6 7 8 9 10

(not accepted) (very well accepted)

Alternative: Don’t know (0).

1. Question:

**German:** Wie bewerten sie die Kommunikation der durch Tierversuche gewonnen Erkenntnisse an die allgemeine Bevölkerung?

*Beispiel: Erprobung von Herzpumpen in Tierversuchen bevor diese am Menschen verwendet werden können; Reanimationsforschung an Tieren um das Überleben nach Herzstillstand zu verbessern*

1 2 3 4 5 6 7 8 9 10

(sehr schlecht) (sehr gut)

Oder: Weiß nicht (0).

**English:** How do you rate the communication of results and knowledge gained from animal experiments to the general Public?

*Example: Testing of heart pumps in animal experiments before use in humans. Resuscitation research on animals to improve survival of cardiac arrest.*

1 2 3 4 5 6 7 8 9 10

(very poor) (very good)

Alternative: Don’t know (0).

1. Question:

**German:** Sollte die allgemeine Bevölkerung mehr über die Anwendung, Nutzen und gesetzliche Grundlage von Tierversuchen informiert werden?
*Beispiel: Entwicklung neuer Therapien für Krebserkrankungen am Tiermodell, bevor potentiell toxische Substanzen am Menschen angewandt werden dürfen.*

1 2 3 4 5 6 7 8 9 10

(überhaupt nicht) (auf jeden Fall)

Oder: weiß nicht

**English:**  Should the general public receive better information on the application, use and legal basis of animal experiments?

*Example: Development of new therapies for cancer in animal models, before potentially toxic drugs are applied to humans.*

1 2 3 4 5 6 7 8 9 10

(definitely not) (absolutely)

Alternative: Don’t know (0).
