# Supplementary material for: Acceptance of animal research in our science community
Source: F1000Res. 2016 Jul 8;5:282. Originally published 2016 Mar 4. [Version 2] doi: 10.12688/f1000research.8169.2 (PMC4866631; doi:10.12688/f1000research.8169.2)
Supplement: Peer review history

# Peer review: Referee response for version 1

**DOI:** 10.5256/f1000research.8786.r14281

**Published:** 2016-06-10

**Authors:** David Bernhard

**Affiliations:** 1 Cardiac Surgery Research Laboratory, Department of Cardiac Surgery, Medical University of Innsbruck, Innsbruck, Austria

The study is well performed and of high interest for the scientific community. Similar studies in the general population would be very valuable and could help in the discussion and communication of the need for animal experiments.

I have read this submission. I believe that I have an appropriate level of expertise to confirm that it is of an acceptable scientific standard.

# Peer review: Referee response for version 1

**DOI:** 10.5256/f1000research.8786.r13710

**Published:** 2016-05-11

**Authors:** David B. Lumenta

**Affiliations:** 1 Division of Plastic, Aesthetic and Reconstructive Surgery, Department of Surgery, Medical University of Graz, Graz, Austria

Survey among doctoral students and staff members (1 university) on their own and general public's perception of animal experiments with 6.38% response rate. The presented questionnaire was general without requiring too much detail from respondents, which I found sufficient.

The results are similar to previous research (as cited by the authors), the used methodology sound, and the conclusions, notably the need for a more systematic review among institutions in Europe on this topic, well balanced.

I have read this submission. I believe that I have an appropriate level of expertise to confirm that it is of an acceptable scientific standard.

# Peer review: Referee response for version 1

**DOI:** 10.5256/f1000research.8786.r13344

**Published:** 2016-04-26

**Authors:** Thomas Butts

**Affiliations:** 1 School of Life Science, University of Liverpool, Liverpool, UK

**General comments**

This report communicates the details of a small, but not insignificant survey of doctoral students’ and professional scientists’ from the Medical University of Vienna attitudes towards animal research. The findings, perhaps not surprisingly, reveal strong support for animal research within the life science research community at this single institution, but importantly also highlight an awareness of the short-comings of communicating this necessity to the public in the light of recent political movements in opposition to research using animals. The data in reference to the use of a given medical treatment without previous testing on animals is particularly interesting and will contribute much to the debate. My sole concern with this research is the generality of the questions. For example, a distinction ought to be made in reference to the type and severity of treatment in the survey. Nevertheless, the data presented will contribute to public (and scientific) debate.

The authors are correct in identifying the need for a more systematic survey amongst European life science institutions and professional groups that will inform this important area of public debate.

**Specific comments**

**Introduction**

Line 1: ‘’…still debated highly controversial and lately…’’ should read ‘’…still debated, highly controversial, and lately has attracted…’’

**Methods**

Paragraph 2, line 1: ‘’resulting’’ should be changed to ‘’representing’’

Line 10: ‘’the’’ should be deleted

I have read this submission. I believe that I have an appropriate level of expertise to confirm that it is of an acceptable scientific standard.
